# Impact of Symptomatology, Clinical and Radiological Severity of COVID-19 on Pulmonary Function Test Results and Functional Capacity during Follow-Up among Survivors

**DOI:** 10.3390/jcm13010045

**Published:** 2023-12-21

**Authors:** Ewa Pietruszka-Wałęka, Michał Rząd, Magdalena Żabicka, Renata Rożyńska, Piotr Miklusz, Emilia Zieniuk-Lesiak, Karina Jahnz-Różyk

**Affiliations:** 1 Department of Internal Medicine, Pneumonology, Allergology and Clinical Immunology, Military Institute of Medicine—National Research Institute, Szaserów 128, 04-141 Warsaw, Poland; 2 Department of Radiology, Military Institute of Medicine—National Research Institute, Szaserów 128, 04-141 Warsaw, Poland

**Keywords:** SARS-CoV-2, COVID-19, pulmonary function test, spirometry, diffusing capacity, plethysmography, 6-min-walk test, quality of life, long COVID, post-COVID syndrome

## Abstract

One of the most commonly observed complications after COVID-19 is persistent pulmonary impairment. The aim of this study was to evaluate the impact of individual factors during the acute phase of COVID-19 on subsequent pulmonary function test results. The study involved 46 patients who were admitted to hospital due to respiratory failure caused by SARS-CoV-2 and who were assessed during follow-up visits at 3 and 9 months after discharge. Patients were divided into two subgroups according to the severity of respiratory failure. The severe group included patients requiring mechanical ventilation or HFNOT. The results of the study showed that a severe course of the disease was associated with a lower FVC and a higher FEV1/FVC ratio 3 months after discharge (both *p* < 0.05). In addition, it has been revealed that the length of hospitalization is a factor that negatively impacts the FEV1, FVC and TLC values measured at follow-up after 3 months. Furthermore, the obtained results identify the presence of cough in the acute phase of the disease as a factor having a positive impact on several PFT parameters (especially the FEV1/FVC ratio) as well as the 6MWT outcome after 3 months. The FVC improved significantly (*p* < 0.05) between the follow-up visits. The findings may indicate that COVID-19-induced respiratory dysfunction is usually temporary and spontaneously resolves during recovery. Recovery is slower in those who required more intensive oxygenation. The results of this study may be useful in identifying patients who require more intensive and longer rehabilitation after COVID-19.

## 1. Introduction

Severe acute respiratory syndrome coronavirus 2 (SARS-CoV-2) was identified for the first time in 2019 in the city of Wuhan, China. Spreading progressively to other regions, the pathogen caused the largest global pandemic of the 21st century [1,2,3]. The disease caused by SARS-CoV-2 was named Coronavirus Disease-2019 (COVID-19) by the World Health Organization (WHO) [1].

SARS-CoV-2 is a positive-sense, single-stranded RNA virus that belongs to the family of *Coronaviridae* [1]. Infection in humans is transmitted by respiratory droplets [4]. The virus invades human cells using angiotensin-converting enzyme 2 (ACE-2) receptors [2,4,5,6]. It can attack cells of different tissues that belong to various systems—including the respiratory, cardiovascular, nervous, gastrointestinal, urinary, endocrine and other systems [6,7,8,9,10]. Acute respiratory tract infection remains the most common manifestation of the coronavirus disease (observed especially in the early days of the pandemic, before the introduction of the vaccine) [11].

According to WHO, by the end of July 2023, more than 750 million cases of SARS-CoV-2 infection were confirmed worldwide, including more than 11 million fatal cases [12]. In Poland, from March 2020 until the end of July 2023, more than 6 million cases were confirmed, including almost 120,000 deaths [13]. According to Kanecki et al., the median age of patients requiring hospitalization due to COVID-19 in the Polish population was 60. Statistically, men were more frequently affected, regardless of age. Patients living in urban areas were also more likely to require hospitalization compared with those living in rural areas. Risk factors for developing severe COVID-19 included primarily age and comorbidities, such as cardiovascular diseases, hypertension, diabetes, COPD (chronic obstructive pulmonary disease), chronic kidney disease, and cancer, as well as being of male gender [14].

The course of SARS-CoV-2 infection can vary widely, from asymptomatic through to mild respiratory tract infection and interstitial pneumonia (often leading to respiratory failure) to a critical course with multi-organ failure requiring mechanical ventilation and intensive care [1,9,15].

The most common symptoms in the initial phase of the disease include fever, cough, myalgia and dyspnea, which worsen with the progressive involvement of the lung parenchyma due to inflammatory lesions [12,16,17]. Computed tomography (CT) of the chest shows bilateral ground-glass opacities with a peripheral distribution. Diffuse parenchymal consolidations, reticular lesions, interlobular septal thickening, crazy-paving patterns and many more may also be present [18].

The spread of the COVID-19 pandemic and the accompanying increase in the number of patients requiring medical care forced rapid changes to the organization of public health services [19,20]. In order to provide emergency care for COVID-19 patients and to limit the transmission of the infection, in many regions of the world, existing wards or entire hospitals were transformed into units dedicated exclusively to the treatment of SARS-CoV-2-infected patients. Temporary hospitals were established, and many different changes were introduced to reorganize hospital wards and outpatient healthcare, including methods based on telemedicine [21,22,23].

Currently, with the most intense phase of the pandemic over, the focus should be placed on the long-term consequences of COVID-19. These may persist for many months and involve multiple organs belonging to different systems, affecting patients in different age groups and with a different severity of symptoms during the acute phase of the disease [9,24,25]. The presence of symptoms lasting more than 12 weeks after the onset of SARS-CoV-2 infection, that cannot be otherwise explained, was named post-acute COVID-19 syndrome (PACS) [24].

Symptoms persisting for such a long period may have a significant impact on the health and everyday functioning of the convalescents. Considering the large number of patients who have experienced pneumonia caused by SARS-CoV-2 in recent years, PACS symptoms may be a challenge and a burden for health systems in many countries [25].

According to recently published studies, the most common symptoms that persist after COVID-19 include general weakness, shortness of breath and cough. Relatively often, patients also report memory and concentration difficulties and palpitations [24,25,26,27,28,29,30,31].

One of the most widely studied aspects of PACS is respiratory function deterioration. Several studies reporting post-COVID respiratory impairment have been published so far. The most common abnormality found in PFTs, which are performed at different time intervals from the onset of the disease, is a reduced DLCO value [9,27,29,30,31,32,33,34,35,36,37,38,39], followed by restrictive disorders [27,30,31,32,33,34,35,37,38,39].

Most authors have identified the severity of the course of COVID-19 as a factor which negatively affects respiratory function, as measured at follow-up visits. However, there are some differences in the reported results between the studies [29,30,33,35,37,38]. For example, contrary to the majority of studies that show a correlation between the disease severity and both a reduced DLCO and restrictive abnormalities, a study by Qin et al. demonstrated a significant effect of the severity of COVID-19 on DLCO values after 3 months, while no effect of disease severity on other lung function parameters, i.e., TLC, FVC and FEV1, was observed [27]. In addition, most authors have reported an improvement in respiratory parameters during the recovery period [30,31,32,39,40,41]. However, in some publications, a progressive deterioration of lung function is mentioned [42]. In a study by Zhang et al., an increase in FVC was observed only during the first year after COVID-19, which was then followed by a decrease, whereas for TLC and DLCO values, a continuous decrease was observed, both within the first year after infection as well as during the second year of follow-up [42].

Moreover, many authors have recently drawn attention to the impact of COVID-19 on the quality of life of the recovered. It has been shown that 3 months after the disease, more than 50% of convalescents score below the population norm on the EQ5D questionnaire, while one year after a SARS-CoV-2 infection, patients have greater problems with mobility, the incidence of pain/discomfort, as well as anxiety and depression compared with the control group [29,32].

As mentioned before, a number of studies that confirm persistent pulmonary impairment following SARS-CoV-2 pneumonia have already been published. In contrast, the aim of our research was to investigate the influence of disease severity on the degree of pulmonary function reduction and to identify other factors that may influence pulmonary impairment. This could be helpful in profiling patients with a relatively mild course of COVID-19, who nevertheless require prolonged follow-up. A further goal of this study was to assess whether reduced respiratory function as measured in PFTs results in a deterioration of quality of life and impairment of patients’ daily functioning.

Almost all patients included in our study group suffered respiratory failure during the acute phase of the disease (and were hospitalized for that reason). In order to assess the impact of disease severity on PFT results, patients were divided into two subgroups—those with a severe versus those with a non-severe disease course. A severe course was defined as respiratory failure requiring high oxygen supplementation (>15 L/min) via high-flow nasal oxygen therapy (HFNOT) or mechanical ventilation.

## 2. Materials and Methods

### 2.1. Patients and Methods

The study group consists of 46 patients (48% females, 52% males) who were admitted to the Military Institute of Medicine—National Research Institute, in Warsaw due to respiratory failure caused by COVID-19 between October 2020 and June 2021. All patients gave voluntary informed consent to participate in the study. The study protocol was submitted to the local Bioethics Committee, which issued approval no. 25/WIM/2021.

The results of examinations performed during the acute phase of infection in accordance with available recommendations were compared with previously scheduled examinations performed during follow-up visits. Patients were followed up 3 and 9 months after their hospital discharge.

At the follow-up visit, each patient underwent radiological examinations, i.e., HRCT (high-resolution computed tomography), and, in selected, medically indicated cases, contrast-enhanced chest CT or CT pulmonary angiogram. Pulmonary function tests (PFTs), i.e., spirometry, plethysmography and the determination of the diffusing capacity for carbon monoxide (DLCO) were also performed, as well as 6-min walking tests. In addition, patients were asked to complete a questionnaire regarding the occurrence of selected symptoms during the acute phase of COVID-19 and at the time of follow-up visits, as well as a questionnaire assessing the quality of life at both follow-up appointments (EuroQol-5D (EQ-5D) + Visual Analog Scale (VAS)) [43]. In the analysis, appropriate coefficients from the parameterization model of the EQ-5D questionnaire adjusted for the Polish population were used [44].

Pulmonary function tests were performed with the JAEGER MasterScreen Body/Diffusion device (~230 V, 50/60 Hz, 508 VA, IP20). The following parameters were included in the analysis: FEV1 (forced expiratory volume in 1 s), FVC (forced vital capacity), FEV1/FVC ratio, TLC (total lung capacity) and DLCO (diffusing capacity for carbon monoxide). The results are presented as a percentage of the predicted values according to the Global Lungs Initiative (GLI-2012) [45]; the FEV1/FVC ratio is also presented as a crude FEV1/FVC ratio.

Radiological imaging findings were assessed by an experienced radiologist. The severity of pulmonary parenchymal lesions during the acute phase of COVID-19 and at subsequent checkpoints was assessed using the CT severity score (CT CSS), with scores ranging from 0 to 25 depending on the severity of the lesions typical for COVID-19; 0 to 5 points for each of the five lung lobes [46].

The 6MWT was performed by qualified personnel according to the guidelines of the Polish Association of Lung Diseases [47]. The results achieved by the patients were presented as a percentage of the predicted value (estimated according to the formula proposed by Gibbons) [48]. In addition, the severity of dyspnea was rated before and after exercise using Borg’s 10-point scale [47].

The severity of the disease course of COVID-19 was also included in the analysis. The study group was divided into two subgroups: patients with a severe course of infection and the remaining patients. A severe course was identified in those patients requiring oxygen therapy with a flow of >15 L/min via high-flow nasal oxygen therapy (HFNOT) or mechanical ventilation (invasive or non-invasive).

The presence of chronic respiratory disease at the onset of COVID-19 (COPD, asthma or other), patients’ age at the time of hospital admission, the presence of a cough during the acute period of the illness, the number of symptoms present at the time of hospital admission (such as fever, cough, shortness of breath, weakness, muscle aches, chills, headache, diarrhea, nausea, sore throat, smell/taste disorders) and treatment administered were also analyzed among the study group.

### 2.2. Statistical Analysis

The analysis was performed using the R software (version 4.2.3), Statistica (TIBCO, version 13) and MS Excel (version 2311). Quantitative variables were presented as means and standard deviations or medians and quartile ranges for data with normal or non-normal distribution, respectively. Nominal data were presented as numbers and percentages. The Shapiro–Wilk test was used to verify a normal distribution. Groups were compared using the Student’s *t*-test or Mann–Whitney U test for data with normal or non-normal distribution, respectively. For dependent data, the Student’s *t*-test or the Wilcoxon signed-rank test were used for parametric and non-parametric data, respectively. To evaluate the effect of individual factors on PFT outcomes, multiple regression was used. A two-sided *p* value lesser than 0.05 was considered statistically significant.

The required sample size was calculated for the FVC (% of predicted value) between severe and non-severe COVID-19 patients using the G-Power software and data from the Zhang et al. study [42], assuming a type I error probability of 5%, a test power of 80% and an allocation factor of 0.5 (n severe cases/n non-severe cases). The calculated sample size was estimated at 38 patients.

## 3. Results

### 3.1. Results of Pulmonary Function Tests in Relation to the Severity of the COVID-19 Acute Phase

The study group consisted of 46 patients (48% females, 52% males). The median age of the patients was 63 years (IQR 53–69). The median length of hospital stay was 20 days (IQR 11–31 days).

Patients were divided into two subgroups based on the severity of the acute phase of COVID-19—a severe group and a non-severe group, depending on oxygen requirements (definition above). The median age of the first group was 64 years (IQR 57–66), while that of the second was 63 years (IQR 57–72). The median length of hospital stay for the group with severe and non-severe COVID-19 was 32 days (IQR 23–48 days) and 12 days (IQR 10–20 days), respectively.

#### 3.1.1. Results of Pulmonary Function Tests 3 Months after Hospital Discharge

We found that patients with a severe course of the disease achieved statistically significantly lower FVC values (%) in spirometry tests (*p* < 0.05) at the first follow-up visit 3 months after their discharge (Table 1). In addition, they achieved statistically higher FEV1/FVC index values (expressed both as the percentage of the predicted value and as a crude FEV1/FVC ratio; both with *p* < 0.005). The difference between the subgroups in TLC as measured by plethysmography was close to the threshold for statistical significance (*p* = 0.058), with lower values found in patients with severe COVID-19. There were no significant differences in FEV1 and DLCO values between the two subgroups (Table 1).

#### 3.1.2. Results of Pulmonary Function Tests 9 Months after Hospital Discharge

There were no significant differences between the severe and non-severe COVID-19 subgroups in pulmonary function tests performed at the second follow-up visit 9 months after hospital discharge.

### 3.2. Influence of Plasma Administration in the Acute Phase of the Disease on Pulmonary Function Test Results at Follow-Up Visits

The effect of the administration of convalescent plasma (indicated in the WHO guidelines issued during the early period of the pandemic) on pulmonary function tests was also investigated. There was no significant association between the use of plasma and pulmonary function tests for the analyzed parameters.

### 3.3. Influence of Symptoms Present during the Acute Phase of the Disease on the Results of Pulmonary Function Tests

**Figure 1:**
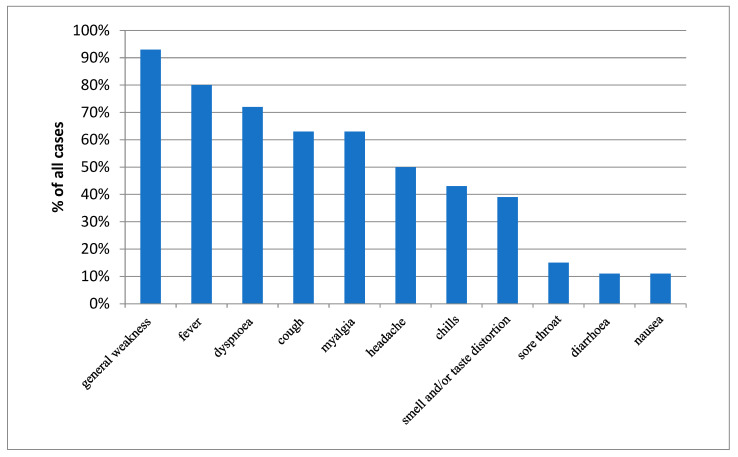
Prevalence of selected symptoms present during the acute phase of COVID-19.

Another analyzed area was the impact of symptoms present during the acute phase of the disease on the results of PFTs. The presence of symptoms was assessed using a questionnaire that patients were asked to complete at each follow-up visit. The questionnaire covered the following symptoms: general weakness (present in 93% of all patients), fever (80%), dyspnea (72%), cough (63%), muscle pain (63%), headache (50%), chills (43%), smell and/or taste disorders (39%), sore throat (15%), diarrhea (11%), and nausea (11%) (Figure 1).

There was no significant correlation between the number of symptoms present during the acute phase of the disease and the results of the PFTs.

Additional analyses were performed for the occurrence of cough during the acute phase of the disease, this symptom being one of the most characteristic of COVID-19 (Table 2).

#### 3.3.1. Association between the Incidence of Cough in the Acute Phase of COVID-19 and PFT Results after 3 Months

It was revealed that the presence of cough during hospitalization was associated with a significantly higher FEV1/FVC ratio, expressed as both the FEV1/FVC crude ratio and as a percentage of the predicted value (*p* = 0.01 and *p* < 0.05, respectively). Furthermore, the presence of cough correlated with a significantly higher DLCO score obtained at the first follow-up visit (*p* < 0.05).

#### 3.3.2. Association between the Incidence of Cough in the Acute Phase of COVID-19 and PFT Results after 9 Months

The presence of cough during the acute phase of COVID-19 was correlated with a higher FEV1/FVC ratio measured at the second follow-up visit 9 months after hospital discharge. This was found for the FEV1/FVC ratio expressed both as a crude ratio (*p* < 0.05) and as a percentage of the predicted value (*p* < 0.05).

### 3.4. Differences between the Results of Pulmonary Function Tests Obtained at the First and Second Follow-Up Visits

**Figure 2:**
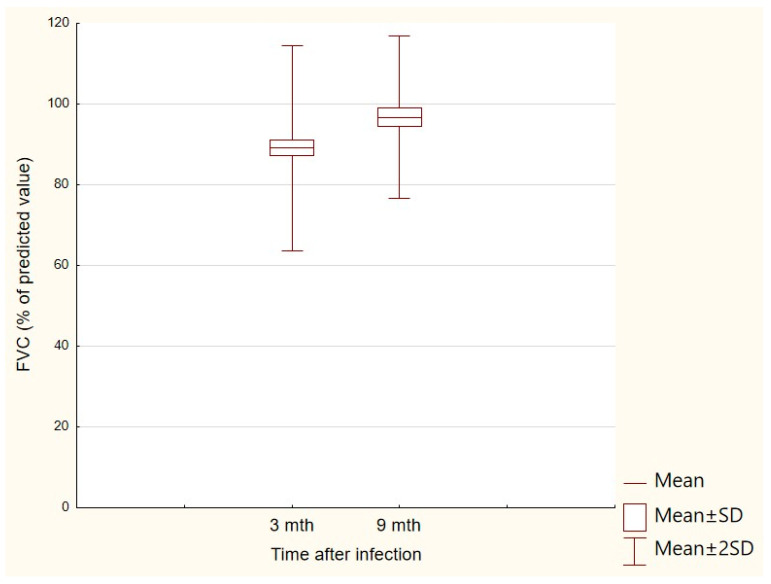
FVC (% of predicted value) results obtained at the first and second follow-up visits.

Changes in the results of various parameters included in PFTs performed at both follow-up visits were also analyzed. The change in the obtained results was significant only for the FVC parameter (*p* < 0.05)—after 3 months, the mean was 94.3% for the FVC (SD: 10.5%), while after 9 months, the mean was 96.8% (SD: 10.1%); *p* < 0.05 (Figure 2).

### 3.5. Effect of Individual Factors on PFT Outcomes

To assess the impact of individual factors on PFT outcomes, regression analysis was performed using data on symptoms reported in the acute phase of COVID-19, gender, age, number of days of hospitalization, severity of the course of the disease, treatment administered and imaging findings during infection (CCS). The proposed significant predictive models are presented below.

#### 3.5.1. Effect of Individual Factors on PFT Outcomes 3 Months after Hospitalization

- FEV1 (% of predicted) = 99.258 − 0.335 × (days of hospitalization); adjusted R^2^ = 0.16, *p* < 0.005
- FVC (% of predicted) = 99.521 − 0.432 × (days of hospitalization); adjusted R^2^ = 0.35, *p* < 0.001
- FEV1/FVC (% of predicted) = 96.054 − 4.879 × (male gender: 1—Yes, 0—No) + 8.919 × (cough in acute phase: 1—Yes, 0—No) + 6.897 × (severe COVID-19: 1—Yes, 0—No); adjusted R^2^ = 0.41, *p* < 0.001
- DLCO (% of predicted) = 87.995 − 1.125 × (CCTS) + 9.586 × (cough in acute phase: 1—Yes, 0—No); adjusted R^2^ = 0.17, *p* < 0.01
- TLC (% predicted) = 91.030 + 0.300 × (age) − 0.536 × (days of hospitalization) − 11.528 × (myalgia in acute phase: 1—Yes, 0—No); adjusted R^2^ = 0.40, *p* < 0.001

#### 3.5.2. Effect of Individual Factors on PFT Outcomes 9 Months after Hospitalization

- FEV1 (% of predicted): no significant model
- FVC (% of predicted): no significant model
- FEV1/FVC (% of predicted) = 92.628 + 8.035 × (cough in acute phase: 1—Yes, 0—No) + 7.267 × (smell/taste distortion in acute phase: 1–Yes, 0–No) − 12.407 × (sore throat in acute phase: 1—Yes, 0—No); adjusted R^2^ = 0.54, *p* < 0.005
- DLCO (% of predicted): no significant model
- TLC (% of predicted) = 116.000 − 25.313 × (treatment with tocilizumab: 1—Yes, 0—No) − 19.188 × (general weakness in acute phase: 1—Yes, 0—No); adjusted R^2^ = 0.62, *p* < 0.001

### 3.6. Effect of Individual Factors on 6MWT Results 3 Months after Hospitalization

To assess the impact of individual factors on 6MWT results 3 months after hospitalization, regression analysis was performed using data on symptoms reported in the acute phase of COVID-19, gender, age, number of days of hospitalization, severity of the course of the disease, treatment administered and imaging findings during infection (CCS), as well as on results of the PFTs performed 3 months after hospitalization. The proposed significant predictive models are presented below.

- 6MWT distance (% of predicted) = 38.382 + 17.878 × (male gender: 1—Yes, 0—No) − 9.863 × (fever in acute phase: 1—Yes, 0—No) + 12.476 × (cough in acute phase: 1—Yes, 0—No) + 0.237 × (TLC after 3 months (%predicted)); adjusted R^2^ = 0.54, *p* < 0.001

### 3.7. Relationship between Pulmonary Function Test Results and Patients’ Quality of Life

Another analyzed relation was the influence of PFT results on patients’ quality of life. There was no significant correlation between the results of pulmonary function tests (regarding assessed parameters) and the patients’ quality of life in any of the domains of the EQ-5D questionnaire or in the VAS score at both checkpoints.

## 4. Discussion

The COVID-19 pandemic has left its mark on the functioning of the entire global healthcare system. Apart from the organizational difficulties caused by the huge inflow of patients requiring immediate hospital care, an additional burden is the long-term consequences of the infection [19,20,25]. These complications can affect various systems; however, due to the tropism of the virus for pulmonary cells, one of the most common is the impairment of respiratory function that is observed in convalescents [27,34]. Moreover, late complications that persist for many months can have a significant negative impact on patients’ quality of life, both in the mental and physical spheres [29,42].

Our study aimed to identify specific potential factors that may influence the prevalence and long-term persistence of respiratory function complications of interstitial pneumonia caused by COVID 19. This could be a helpful indication for clinicians in identifying patients with COVID-19 who will require prolonged follow-up and intensive rehabilitation.

The obtained results revealed that patients with a severe course of COVID-19 had significantly lower FVC values (mean: 82.9% of predicted) at the first follow-up (3 months after hospital discharge) than patients with a non-severe course (mean: 92.5% of predicted). This relationship was not observable in the controls at 9 months after discharge. In addition, the difference in the achieved TLC (% of predicted) measured after 3 months was also close to statistical significance during this period (lower in patients with a severe course). This relationship would perhaps be observed in a study group with a larger sample size. This may indicate the persistence of residual inflammatory lesions that lead to a decrease in the effective alveolar ventilation. Moreover, this thesis is supported by the fact that at the second follow-up (6 months later), no such correlation was shown, which may reflect the withdrawal of residual changes in the pulmonary parenchyma over time and an associated increase in the TLC. In several studies, similar results have been reported that indicate the severity of COVID-19 as a risk factor for restrictive ventilation patterns, expressed as reduced FVC and TLC values in follow-up measurements at different intervals after the onset of the disease—after 2 [33], 4 [37], 6 [49] and even 12 months [30]. On the other hand, in the Chinese study by Qin et al., despite the observed reduction in the % of predicted TLC and % of predicted FVC (in 10% and 21% of patients, respectively) at the 3-month follow-up, there was no correlation between the reduction level and the severity of COVID-19 [27]. Therefore, this subject requires an extensive analysis involving a larger group of survey participants.

It is worth noting that patients with a severe course obtained statistically higher FEV1/FVC index values at the first follow-up visit. However, this was not a result of differences in the achieved FEV1 values (88.8% of predicted for the severe and 91.9% of predicted for the non-severe course) but rather of the previously mentioned reduction in FVC in this group of patients. These results may suggest that the ventilation abnormalities observed after COVID-19 are caused by the restriction associated with the presence of residual pulmonary parenchymal lesions rather than by airflow impairment. Similar results were reported in a Swiss study [37], in which patients with a severe/critical course of COVID-19 (defined as saturation < 90%, tachypnea RR > 30/min) or a critical course (i.e., ARDS, sepsis, septic shock, multi-organ failure) had significantly lower TLC, FVC, FEV1 and DLCO values at follow-up after 4 months compared with patients with a mild-to-moderate course. Similar to our findings, that study, reported an increased FEV1/FVC ratio in patients who went through an infection that had been classified as severe/critical.

Contrary to the results of other publications [27,29,33,35,37,42], our study showed no significant impact of disease severity on the DLCO value. This may be due to the implementation of different criteria for the disease severity assessment. All patients included in this study were diagnosed with interstitial pneumonia, and the majority of them were in respiratory failure, which means that the study did not include asymptomatic patients or patients with mild symptoms, who would probably have achieved better results in pulmonary function tests. In both subgroups of patients (severe and non-severe), the mean DLCO value at the first follow-up visit was below 80% of the predicted value (73.9% of predicted for the severe group and 79.8% of predicted for the non-severe group). Although the results of the majority of studies are coherent, indicating decreased lung-diffusing capacity as the most commonly observed abnormality in PFTs post-COVID-19, they differ significantly in the dynamics of DLCO changes during follow-up (increase/decrease/constant). For example, Wu X et al. observed a general gradual improvement in DLCO values at subsequent follow-ups (at 3, 6 and 12 months), while a persistent decrease in DLCO value (<80% of predicted) was seen in 33% of patients 12 months after the disease [31]. This may indicate the presence of additional, as-yet-unidentified factors affecting the diffusion capacity of the lungs after SARS-CoV-2-induced interstitial pneumonia.

An analysis of changes in the pulmonary function tests between the two checkpoints for the whole study group revealed a statistically significant difference only for the FVC parameter, which, at the 9-month follow-up, was significantly higher than at the first follow-up carried out 3 months after hospital discharge. This finding supports the thesis that a decrease in effective lung volume takes place, which then increases as the healing process progresses. Similar results were obtained in a recently published study by Chaiwong et al. [50], which showed that percentages of the predicted FVC and FEV1 values were significantly lower in post-COVID patients compared with healthy controls, which then improved during follow-up in the post-COVID group. Guziejko et al. found no significant differences in PFT results between post-COVID patients and healthy controls at follow-up 6 months after discharge [28]. Some other studies have reported similar results regarding an increase in the % predicted FVC during follow-up [30,32,39,42] although the study by Zhang et al. highlights that this parameter increases between the 6th and the 12th month of follow-up and then gradually decreases during the second year of observation [42].

Another area of research that evaluated the impact of the number of symptoms present in the acute period of the disease on the results of pulmonary function tests showed no significant correlations (both at 3 and 9 months after discharge). However, after dividing patients into two subgroups based on the presence or absence of cough, it was found that patients with cough in the acute phase of the disease had a higher FEV1/FVC ratio at both follow-up visits and a higher DLCO value at the first follow-up visit. It is difficult to clearly identify the reason for this relationship. Perhaps patients with a strong cough reflex had a more efficient airway clearance of mucus, which could result in better FEV1 measurements. It is also possible that these patients had a more severe disease course which resulted in greater FVC impairment at follow-up. In fact, it may be that both mechanisms coexist and contribute to the observed phenomenon. In addition, patients reporting cough during the acute period of the illness had higher DLCO values at the first follow-up. A potential explanation for this phenomenon may be that, in patients with the most severe course of SARS-CoV-2 pneumonia, other symptoms such as dyspnea or impaired consciousness may have been so severe that they overshadowed the cough, which was therefore not reported. Our results, however, refer only to the presence of cough in the acute phase of the disease. In contrast, the presence of a chronic cough 6 months after COVID-19 has been proven by Guziejko et al. to be a negative factor in the results of control PFTs (regarding FEV1, FVC, FRC, TLC and DLCO) [28].

While investigating the influence of individual factors on PFTs, we found some interesting dependencies. The results indicate that both FEV1 and FVC (% of predicted) values measured 3 months after hospital discharge are inversely proportional to the duration of hospitalization (expressed in days). However, it should be noted that for the FVC value, the proposed model is moderately strong (R^2^ = 0.35), while for FEV1, it shows a weak relationship (R^2^ = 0.16), which indicates the existence of other unexplored factors affecting these values.

The study by Eberst et al. analyzed factors influencing the DLCO value after 3 months. Its findings indicate that there are three factors negatively influencing the DLCO score—one of them being the length of stay in the intensive care unit. Interestingly, both our study and the aforementioned study included patients with a relatively severe course of the disease (respiratory failure and ICU stay, respectively), which may suggest a possible correlation in this group of patients between the length of hospitalization and long-term negative consequences regarding respiratory mechanics and gas exchange. This subject, however, requires further targeted investigation [41].

While analyzing the effects of various factors on FVC and FEV1 values 9 months after discharge, we found no significant correlation. Considering the significant improvement in FVC demonstrated in our study group between 3 and 9 months of follow-up, as well as the improvement over time in many ventilation parameters described in studies by other authors, it is most likely that the patients’ PFTs improved or even normalized at this timepoint regardless of the degree of previous impairment.

Another relationship studied was the effect of individual factors on the value of the % of predicted FEV1/FVC. Our results indicate that this parameter depends on gender, the presence or absence of cough and the severity of the acute phase of COVID-19 (R^2^ = 0.41). The presence of cough and the severity of illness are positive predictors, while male gender appeared to negatively affect the predicted FEV1/FVC ratio, although there was no direct difference in the severity of the course between the sexes. Based on WHO data, there are about 4 times more smokers among men than among women [51]. Despite the lack of gender differences in terms of obstructive disease present at the time of onset, there may have been a higher prevalence of undiagnosed COPD or subclinical obstructive changes among men, which then translated into a decrease in the FEV1/FVC ratio. In contrast, cough and severity of illness can decrease the FVC, which consequently increases the FEV1/FVC ratio. Another possible explanation for this phenomenon is a more efficient airway clearance in patients with cough present in the acute phase of the disease, which might result in a higher FEV1 score.

One of the most interesting correlations that our study has revealed is the relationship between the DLCO value measured 3 months after discharge and the intensity of the lesions in the baseline chest CT, as well as the presence of cough in the acute phase of the disease (R^2^ = 0.17). The obtained DLCO is inversely proportional to the intensity of CT lesions, while the presence of cough positively affects its value. No such correlation was shown for results obtained 9 months after discharge. Similarly, a study by Qin et al. reported a negative correlation between the severity of lung lesions reflected in CT CSS scores and DLCO values at 3 months [27]. This result may reflect the impairment of gas exchange caused by inflammation (infiltrations of the parenchyma and interstitial tissue of the lungs) manifested as areas of ground-glass opacities and septal thickening in initial chest CTs. The lack of correlation between the severity of CT lesions in the acute phase of the disease and DLCO values after 9 months may, in turn, suggest an improvement in gas exchange with a progressive resolution of the residual inflammatory lesions. Likewise, a study by Wu X et al. [31] found no significant correlation between lesion severity at the initial chest CT and the DLCO measured 12 months after COVID-19.

The lack of correlation between the other assessed ventilation parameters and the severity of the changes in the initial chest CT confirms that the development of inflammatory lesions in the pulmonary parenchyma leads to respiratory failure mainly via the mechanism of impaired gas exchange.

The positive effect of cough on DLCO values remains coherent with the previously described correlation, which indicated that in the group of patients presenting cough in the acute phase of the disease, higher DLCO values were obtained at the first follow-up visit.

Another relationship found in the PFT analysis after 3 months concerns the TLC value. Our results indicate that patient age, duration of hospitalization and the presence of myalgia in the acute phase of the disease are factors affecting the % of predicted TLC measured at the first follow-up. Interestingly, patient age is a positive predictor of the TLC value, while the other two factors affect it in a negative way. Perhaps, it is due to the higher prevalence of emphysema in the older patient group and the associated increase in residual volume (RV); however, this was not assessed in this study. It is likely that the duration of hospitalization reflects the degree of lung tissue damage, which leads to worse TLC results obtained at follow-up. It is also possible that the patients who stayed longer in hospital were those who required advanced ventilatory support techniques, which could also negatively affect lung function. Muscle pain, on the other hand, may have led to impaired breathing mechanics, which could be a risk factor for mechanical ventilation, as well as for prolonged oxygen supplementation.

Investigating the effect of individual factors on the FEV1/FVC ratio obtained after 9 months, we found that the presence of cough (similarly to the result obtained after 3 months) and smell/taste impairment are positive predictors for this value, while the presence of a sore throat in the acute phase of the disease affects it negatively. The proposed model is relatively strong (R^2^ = 0.54), indicating that the aforementioned factors influence the FEV1/FVC ratio significantly.

The last regression revealed in our study is the negative impact of treatment with tocilizumab and of the presence of general weakness during the acute phase of the disease on the TLC value after 9 months. This is the strongest predictive model we obtained (R^2^ = 0.62). It seems that it is not tocilizumab itself that has a negative impact on lung capacity. It is probable that this relationship results from the fact that we used this drug in patients who were in an extremely severe clinical condition, with rapid disease progression, who were in the phase of a cytokine storm and, therefore, had a greater impairment of baseline respiratory parameters due to lung tissue damage. Meanwhile, the general weakness reported by patients undoubtedly negatively affected the rehabilitation process, which, in turn, may have resulted in a postponed recovery to their expected PFT values.

Another interesting finding of the study is the correlation between gender, presence of fever and cough in the acute phase of the disease, as well as the TLC score on the 6MWT distance obtained after 3 months (expressed as % of predicted). Male gender, presence of cough during the acute phase of the disease and the % of predicted TLC (after 3 months) are positive predictors of the distance walked in the 6MWT at the first follow-up visit, while the presence of fever during acute COVID-19 has a negative impact on the outcome.

As previously mentioned, male gender and cough (during the acute phase of the disease) have a positive impact on the FEV1/FVC ratio, potentially resulting in better patient physical capacity and, therefore, a better 6MWT score, whereas a positive correlation between total lung capacity and the 6MWT distance has already been reported in patients with interstitial lung disease [52]. In the present study, similarly to the one conducted by Zhang et al. [42], no correlation between disease severity and the 6MWT score (% of predicted) was found, although there are other studies [33,53] that have demonstrated such a relationship.

An interesting finding is the presence of fever in the acute phase, which is a positive predictor of the 6MWT score. A hypothetical explanation for this phenomenon could be that during high fever, there may have been more severe changes in the pulmonary parenchyma as a result of increased inflammation and the occurrence of rhabdomyolysis, which may have translated into worse physical performance in the follow-up period.

The absence of a significant association between the results of pulmonary function tests and the quality of life of the convalescents (assessed by means of both the EQ-5D questionnaire and the VAS scale) may suggest that the degree to which the mechanics of ventilation and gas exchange are impaired does not directly result in a deterioration in the quality of life in terms of mobility, self-care, activity, pain perception and depression, nor does it directly affect the patient’s subjective assessment of their health (via the VAS scale) among the Polish population. This study is not the first to report similar results. The study by Zhang et al. from 2022 also concluded that lung-function deficit did not have a significant impact on the quality of life of survivors [42].

As in any other study, this one has also its limitations, mostly due to the relatively small study group, the presence of respiratory failure in most of the enrolled patients and the simplified classification of disease severity based on oxygen requirements. However, the survey’s advantages minimize these limitations. These advantages include a long follow-up period (up to 9 months after COVID-19), a comprehensive evaluation of pulmonary function parameters (spirometry, plethysmography, diffusing capacity for carbon monoxide), as well as an analysis of both objective (functional tests, chest CT lesions) and subjective (EQ-5D questionnaire, Borg scale, reported symptoms) parameters.

## 5. Conclusions

Despite the limitations, the results obtained in this study allow us to formulate a number of essential conclusions:

- Respiratory dysfunction resulting from COVID-19 is temporary in most cases and spontaneously regresses during the following months of recovery.
- In patients with a high requirement for oxygen supplementation during the acute phase of COVID-19, the process of recovery of respiratory function is slower.
- Higher TLC values and male gender are associated with better overall mobility (assessed by the 6MWT test) in patients who have experienced a SARS-CoV-2 infection.
- The degree of respiratory impairment does not directly correlate with the patients’ quality of life.
- The result of this study may be useful to for identifying patients who require more intensive and longer rehabilitation after a SARS-CoV-2 infection.
- At follow-up after 3 months, the number of days of hospitalization due to COVID-19 was a factor significantly affecting the FEV1, FVC and TLC results.

## Figures and Tables

**Table 1:** Results of pulmonary function tests 3 months after hospital discharge.

Parameter	N—Total	Mean	N—Severe Course	Mean (SD)—Severe Course	N—Non Severe Course	Mean (SD)—Non Severe Course	*p*-Value
FVC (% of predicted)	46	89.1 (12.1)	16	82.9 (11.5)	30	92.5 (12.2)	0.011
FEV1 (% of predicted)	45	89.9 (12.6)	16	88.8 (12.4)	30	91.9 (14.8)	0.548
FEV1/FVC (% of predicted)	45	101.3 (8.9)	16	106.7 (7)	30	98.8 (8.7)	0.002
FEV1/FVC (% FVC)	45	79.5 (7.6)	16	83.7 (5.8)	30	77.4 (7.5)	0.004
TLC (% of predicted)	44	88.9 (16.7)	16	82.6 (12.3)	29	92.3 (17.7)	0.058
DLCO (% of predicted)	44	77.6 (14.9)	16	73.9 (13.5)	28	79.8 (15.5)	0.222

**Table 2:** Pulmonary function test results in relation to the presence of cough during the acute phase of the disease at both follow-up visits.

Parameter	Number (N) of Patients	Mean (SD)	N-Patients with Cough	Mean (SD)—Cough	N-Patients Without Cough	Mean (SD)—without Cough	*p*-Value
after 3 months							
FVC (% of predicted)	46	89.1 (12.7)	29	88.4 (13.6)	17	90.3 (11.3)	0.600
FEV1 (% of predicted)	46	90.8 (13.9)	29	92.9 (14.4)	17	87.3 (12.8)	0.114
FEV1/FVC (% of predicted)	46	101.5 (8.9)	29	104.5 (6.6)	17	96.5 (10.3)	0.010
FEV1/FVC (%FVC)	46	79.6 (7.5)	29	82.2 (5.3)	17	75.2 (8.8)	0.002
TLC (% of predicted)	45	88.9 (16.5)	28	85.6 (14.7)	17	94.3 (18.4)	0.163
DLCO (% of predicted)	44	77.6 (14.9)	27	81.7 (15)	17	71.1 (12.6)	0.026
after 9 months							
FVC (% of predicted)	20	96.8 (10.1)	15	96.8 (11.3)	5	96.6 (5.8)	0.965
FEV1 (% of predicted)	20	97 (13.6)	15	99.6 (13.4)	5	89 (12)	0.088
FEV1/FVC (% of predicted)	20	99.7 (9.7)	15	102.4 (6.8)	5	91.6 (13.2)	0.032
FEV1/FVC (%FVC)	20	78.1 (8.5)	15	80.5 (6.1)	5	70.9 (11)	0.029
TLC (% of predicted)	19	95.2 (11.6)	14	93.4 (10.9)	5	100.2 (13)	0.430
DLCO (% of predicted)	18	87.3 (11.9)	13	90.2 (8.4)	5	79.8 (16.9)	0.182

## Data Availability

The data are available in the repositories of the Military Institute of Medicine in Warsaw and can be made available for legitimate interest after contacting the corresponding author.
